# The War on Cancer: Lessons from the War on Terror

**DOI:** 10.3389/fonc.2014.00293

**Published:** 2014-10-20

**Authors:** Sui Huang

**Affiliations:** ^1^Institute for Systems Biology, Seattle, WA, USA

**Keywords:** war on cancer, stress-response, therapy failure, target-selective drugs, recurrence, somatic evolution

The war metaphor has become commonplace in discussions about our endeavor to find a cure for cancer. But beyond the power of imagery, this analogy offers the opportunity to highlight lessons that warriors of cancer can learn from the warriors of terror: that more efficient killing of the undesired cells may not always be better. Evidence suggesting that drug-stressed and dying cancer cells can drive tumor progression is almost as old as cancer therapy. But like in warfare until recently, the primary focus in cancer therapy has been, and still is, on how to more efficiently kill.

Ever since President Nixon launched the war on cancer in 1971, the war metaphor has offered a convenient communication tool to articulate the ups and downs of our quest for a cure of cancer (1). The repeated use of the war metaphor, however, has directed our attention to the most visible activity of warfare: the use of destructive force which, ironically, military leaders now have recognized as a problem. They have learned their lessons, and here is where the parallels stop: lessons that cancer warriors have yet to learn. Much as indiscriminate carpet bombing has given place to laser-guided bombing of the enemy, so are we replacing broadly cell-killing chemotherapy with modern “target-selective drugs” that can take out with molecular precision the critical proteins required for cancer cell survival and expansion while minimizing collateral damage. Yet these celebrated magic-bullet drugs (2) almost universally fail to eradicate the tumor, which typically relapses after a year or less. And when the tumor recurs, it is more malignant and uncontrollable than before (1).

The current hope is that with even more precise target-selective drugs that circumvent resistance-conferring mutations and by using clever combinations of such drugs, we will ultimately win the long war against the evasive cancer cells (3). Multi-pronged targeted attacks appear plausible in cornering the tumor cells, as we have seen in anti-viral therapy of HIV, and could conquer drug resistance. But cancer cells are not rigid viral particles whose sole means of evasion is genetic mutation. Cancer cells are plastic. The thousands of normal cell types in the human body, all carrying the same genome, manifest an enormous developmental potential. Could cancer cell exploit such inherent developmental plasticity of metazoan cells to adopt, without mutations, new phenotypes to evade treatment (4)? If yes, then, more effective killing to outrun the Darwinian somatic evolution (5) generally thought to drive the development of therapy resistance in tumor cells, might be the wrong answer to therapy failure.

Put more bluntly: what do all cancer drug therapies have in common? Answer 1: they essentially fail to cure cancer. Again, what do all cancer drug therapies have in common? Answer 2: they all seek to perturb, mostly kill, cancer cells. Thus, by pure logics, could killing *eo ipso* be the problem? In fact, all therapies fall into the broad category of killing or arresting tumor cells, directly through cytotoxic or differentiating therapy, or indirectly via inhibition of angiogenesis, altering the tumor bed or enhancing antitumor immunity.

Often, solutions to tough problems come from thinking in a more encompassing category than imagined. Perhaps, questioning the unquestioned notion that “only a dead tumor cell is a good tumor cell” is the first step. This is what cancer biologists and oncologists can learn from lessons learned in the war on terror: to think beyond killing as many enemies as possible.

Targeted bombing in the war on terror has only short-term benefits. Unlike traditional warfare against homogenous armies with soldiers marching in unison, the war on terrorists, and by analogy, the war on cancer cells, is more akin to a guerilla war. It is nearly impossible to specifically kill every single one of the bad guys, not even with precision weaponry. Sure, bombing campaigns are often necessary to avert immediate danger by reducing the numbers of enemy combatants. But the inevitably surviving terrorists will recover, regroup, and adapt, emerging even stronger. This is not the passive “selection” of genetically stronger fighters as modern Darwinists might think, but rather the result of an active response by the lucky survivors of an incomplete attack in a rugged terrain. In the same way, targeted therapy of cancer debulks the tumor, providing urgently needed relief. But like the guerilla combatants in the mountains, cancer cells are too diverse and too disperse, such that high-precision targeted attacks almost always will allow some cancer cells to survive in the tissue. These residual cells will re-emerge as a recurrent tumor – this time resistant to the drug and more malignant than before. But the parallels between the war on terror and the war on cancer end when it comes to the lessons learned. In the course of history, generals have learned a lesson that cancer researchers have not yet learned.

In the wars on terror, military commanders have now recognized that surviving a drone attack energizes people more than any propaganda can (6), creating new fighters, more numerous and fiercer than before and more capable of evading bombs. Our commanders have learned how to wield the sharp double-edged sword that the use of destructive force always is. This sensibility is reflected in the parallel efforts to understand history, tradition, and culture of the enemy and in the campaigns to “win hearts and minds” (7).

Cancer researchers have not yet proceeded to this stage of wisdom. Instead, stuck in the category of thought that “only a dead cancer cell is a good cell” the majority does not pay attention to “history, tradition, and culture” of cancer cells that reach back to our ancestors: tumor cells can actively cope with the stress inflicted by drugs by using the same evolved developmental plasticity that endows a fertilized egg cell with the enormous capacity to develop into a complex multicellular organism without mutating its genome. The same phenotypic plasticity affords stem cells the ability to respond to tissue injury and xenobiotic threats by switching cell states. By contrast, current explanation of the inexorable development of drug resistance is firmly anchored in the orthodoxy of Darwinian evolution (8) that eschews plasticity and espouses rigidity of the genotype–phenotype relationship. It is all about the “survival of the fittest”: the cells that have acquired *by chance* a “resistance-conferring mutation” *before* the therapeutic attack are “*selected for*” by the drug (9, 10). There is no room for phenotype plasticity, let alone the cell’s active defense against near lethal perturbations. Then, all we need are better and more precise weapons, ideally in smartly combined and timed deployment, to neutralize these mutated targets.

But as we now realize, every new target-selective drug faces the development of drug resistance (9, 11–14). In stepping beyond current thinking, we need to embrace some obvious but sidelined facts, which raise the possibility that it is indeed the very act of killing and the intrinsic incompleteness of this act that is the problem: attempts to kill cancer cells in a phenotypically heterogeneous tumor (as they all are, like a guerilla army) with precise target-specific compounds will not kill all target cells, resulting in cell-stress in the surviving cells. Importantly, treatment is not without influence on the cells that we fail to kill: therapeutic intervention represents a massive, near-death cell-stress for the surviving cells. It triggers a phenotype switch to a more primitive and resilient state – a natural, developmentally immature and perhaps evolutionary ancient cell state (15) that is latently present in the theoretical space of gene expression programs. Such immature states epitomize what we now recognize as “cancer stem cell” (15–21). The emergence of resistant cells reflects a non-genetic phenotype switch *induced* by the treatment in *individual* cells and not a cell population shift due to the expansion of a preexisting clone with distinct properties that allowed it to survive (22). What looks like a violation of the Darwinian principle, which prohibits active “adaptation” to a stressor by the individual as source of evolutionary innovation, may actually be the functional reactivation of an ancient program deeply encoded in every cells’ genome that protects them from natural stressors (15, 23). This explains why as sophisticated survival and defense mechanisms as mitogen-independent cell proliferation, hypoxia resistance, DNA damage tolerance, invasion, migration, cell-detoxification, immune evasion, etc. arise in such short time soon after treatment (24). These complex cellular programs are barely explainable by Darwinian selection of random mutations (22). Instead, the physiological, “Lamarckism-like” cellular adaptation permits a short-term expansion, buying time for cells to accumulate genetic mutations in subsequent cell generations that could lead to genetic fixation of these new traits (4, 24). Modern evolution biologists who think beyond Darwin appreciate that phenotypic, non-genetic plasticity may precede Darwinian selection of rigid mutants (25).

But now, it gets worse: the surviving cancer cells, which have endured a non-lethal blow are not only individually strengthened by the toxic molecules but are surrounded by massive cell death of its less lucky neighbors. And dead cells release alarmins (26), a distress signal, which induces cell-defense mechanisms among the surviving cells and also stimulates inflammation (26). These micro-environmental disturbances can trigger a primordial adaptive and reparative response in the tissue (26–28) alongside an increase of “stemness” character of tumor cells (19, 20, 22, 29, 30), much as villagers, including non-combatant civilians who survive a bombing campaign, are alerted by their injured neighbors and strengthened in their own resolve to counter future attacks. Invading inflammatory cells, evolved to repair the injured tissue, secrete growth factors that stimulate angiogenesis as well as tumor cells. Old experiments show that injecting killed cells along with the usual live tumor cells to model an animal tumor dramatically boosts tumor growth (31). Therapy-induced death of some of their own thus triggers a resistance movement of the entire cell community – just as in the war against terrorists.

Summarizing a new principle, we can say that in addition to relying on Charles Darwin’s scheme of “*survival of the fittest*”(32), which operates at longer time scales, tumor cells individually follow Friedrich Nietzsche’s scheme: “*What does not kill me makes me stronger*”(33). This principle permits survival in the short term, winning time for true Darwinian evolution to take effect. Since Nietzsche’s scheme involves a phenotype switch to an ancient, resilient, stress-coping state, it accounts for why recurrent tumors are invariably more aggressive in many more ways than just with respect to the selectable trait of drug resistance. Thus, the inevitable non-genetic cell heterogeneity entails incompleteness of killing, which in turn permits Nietzsche’s principle of emboldening the surviving, stressed cells to take place – amplified by cell–cell communication. This active response of tumor cells as a coherent population makes it hard to eradicate tumor cells and win the war on cancer.

The military has learned its lessons. The recent war on terrorism has illuminated its capability to avoid groupthink and to think in a more encompassing category. Even more, it has *learned to learn* from past failures. This process has been institutionalized by the creation of the aptly named *Center for Army Lessons Learned*, CALL (34).

It is time for a (wake-up) CALL equivalent among cancer warriors. We cancer researchers have not learned, let alone learned to learn, from the war on cancer. The climate of thought at the National Cancer Institute (NCI) for instance is such that it still promotes the view that more killing, with more powerful and more precise weapons, is the way to go. And the tender calls for funding more innovative research by some of its wiser leaders are drowned by the groupthink of the peer-review system that automatically filters out “outside-the-box” applications for research funding. To escape this incestuous system of mutual intellectual confirmation, we need to complement the reflex to scan cancer genomes for drug targets with the reflection on the deeper “culture” of cancer. We need to explicitly ask: Why are we losing the war on cancer? What are the mechanisms of treatment failure? Whence the enormous non-genetic plasticity of tumor cells?

A specific solution may be to minimize the active cellular stress response while attacking the cells. Signaling pathways that mediate this stress response that “makes the non-killed cells stronger,” such as the Wnt pathway (22, 35), could be blocked prior to standard cytocidal therapy – as the equivalent to avoiding provocation and building political support in the countries that host the terrorists.

Cynics may now inject: we are not *winning* the war on terror – how then can the military serve as an example for the war on cancer? We may not be winning but with minimal use of force along with careful politics, we do contain terrorism reasonably well, to a level that is compatible with normal life for most of us. If only in cancer research, we could learn to learn and see that killing may only breed more violent response. Then, as some oncologists envision (36), by taking a gentler approach, such as “maintenance therapy” (37) and by blocking cell-stress response, we may one day safely drag out our patients’ war on their cancer – like we do in the war on terrorism: far into unforeseeable future, turning it into a well-tolerated, barely unnoticed chronic ill.

## Conflict of Interest Statement

The author declares that the research was conducted in the absence of any commercial or financial relationships that could be construed as a potential conflict of interest.
